# Ormeloxifene Versus Norethisterone for Anovulatory Abnormal Uterine Bleeding: An India-Based Systematic Review and Meta-Analysis

**DOI:** 10.7759/cureus.99971

**Published:** 2025-12-23

**Authors:** Saranya Devi, Hemavathy V, Reenaa Mohan, Arularasan J, Preethi Tamilarasan, Suja Xaviar, Saravanan Vaithiyalingam

**Affiliations:** 1 Obstetrics and Gynaecology, Sri Manakula Vinayagar Medical College and Hospital, Puducherry, IND; 2 Community Medicine, Sri Manakula Vinayagar Medical College and Hospital, Puducherry, IND; 3 Paediatrics, Sri Manakula Vinayagar Medical College and Hospital, Puducherry, IND; 4 Pharmacology and Therapeutics, ESIC Medical College and Hospital, Chennai, IND; 5 Community Medicine, Melmaruvathur Adhiparasakthi Institute of Medical Sciences and Research, Melmaruvathur, IND

**Keywords:** abnormal uterine bleeding, aub-o, meta-analysis, norethisterone, ormeloxifene, systematic review

## Abstract

Abnormal uterine bleeding due to ovulatory dysfunction (AUB-O) is a frequent gynecological problem affecting women of reproductive and perimenopausal age. While norethisterone, a synthetic progestogen, has been widely used for medical management, ormeloxifene, a selective estrogen receptor modulator, has recently emerged as a promising alternative.

The objective of this study is to compare the efficacy and safety of ormeloxifene versus norethisterone in the treatment of AUB-O.

A systematic review and meta-analysis were conducted in accordance with Preferred Reporting Items for Systematic Reviews and Meta-Analyses (PRISMA) guidelines and registered with PROSPERO (CRD42024514294). A comprehensive search of PubMed, Google Scholar, Web of Science, and the Cochrane Library was performed up to 2025. Eligible studies included randomized controlled trials and prospective comparative studies evaluating ormeloxifene against norethisterone in women with AUB-O. Outcomes assessed were hemoglobin improvement, reduction in endometrial thickness, and change in Pictorial Blood Assessment Chart (PBAC) score. Data were extracted independently by two authors and analyzed using RevMan 5.4 (The Cochrane Collaboration, Oxford, UK) with a random-effects model.

Five studies comprising 661 women were included (331 received ormeloxifene; 330 received norethisterone). Ormeloxifene significantly improved hemoglobin levels compared with norethisterone (standardized mean difference (SMD) = 0.81 g/dL, 95% CI 0.14-1.47; p < 0.00001; I² = 93%). It was also superior in reducing endometrial thickness (SMD = -0.74 mm, 95% CI -1.48 to -0.01; p < 0.00001; I² = 91%) and lowering PBAC scores (SMD = -1.06, 95% CI -1.25 to -0.87; p < 0.00001; I² = 0%).

This meta-analysis suggests that ormeloxifene is more effective than norethisterone in improving clinical outcomes of AUB-O, with better control of menstrual blood loss, hemoglobin levels, and endometrial thickness. Larger, high-quality multicenter trials with longer follow-up are warranted to confirm long-term safety and efficacy.

## Introduction and background

Abnormal uterine bleeding (AUB) is one of the most commonly encountered gynecological complaints affecting women of all age groups, with a higher incidence in the perimenopausal period [1]. AUB is defined as irregularities in the menstrual cycle involving the parameters of frequency, regularity, duration, and volume of flow outside of pregnancy in reproductive-aged women [2]. It is a challenging problem that affects the physical, social, and mental health of patients, causing deterioration in the quality of personal life due to restlessness and fatigue, and also professional life by decreasing the working capacity of patients [3,4]. Worldwide prevalence of AUB ranges from 4 to 52% [5]. In India, the reported prevalence of AUB ranges from 9 to 30% in premenopausal and reproductive age women and up to 50% in perimenopausal women [6]. The FIGO PALM-COEIN classification identifies structural (polyp, adenomyosis, leiomyoma, malignancy/hyperplasia) and non-structural (coagulopathy, ovulatory dysfunction, endometrial, iatrogenic, and not otherwise classified) causes of AUB [1]. While pattern and causes of AUB differs in different age groups and reproductive statuses, abnormal uterine bleeding due to ovulatory dysfunction AUB(O) is one of the common causes of AUB [7].

While hysterectomy has been a definitive treatment, its potential long-term complications and economic implications have driven interest toward effective medical management of AUB [8]. Norethisterone, a synthetic progestogen, acts by suppressing the endometrial proliferation, regulating cycles, and reducing menstrual flow. However, it is associated with hormonal side effects such as breakthrough bleeding, fluid retention, and increased cardiovascular risk [9]. It carries the risk of adverse effects, including stroke, breast cancer, and dementia. Ormeloxifene, a third-generation selective estrogen receptor modulator (SERM), exerts antiestrogenic effects on the uterus and breast, which slows down proliferation of endometrium while maintaining estrogenic action on bone, vagina, and cardiovascular system [10,11]. It reduces menstrual blood loss by modulating estrogen receptor expression in the endometrium, alleviating dysmenorrhea, mastalgia, and premenstrual symptoms, with the added advantages of convenient weekly dosing, cost-effectiveness, and minimal side effects [6,12].

Several randomized controlled trials have demonstrated that both ormeloxifene and norethisterone are effective in reducing menstrual blood loss, improving hemoglobin levels, and decreasing endometrial thickness, with ormeloxifene showing superior efficacy and better patient compliance [11,13,14]. Considering these findings, a systematic evaluation of both agents is essential to establish the most effective medical management strategy for AUB and to minimize the need for unnecessary surgical interventions.

## Review

Material and methods

This study was designed as a systematic review and meta-analysis and was prospectively registered with PROSPERO (ID 1123511). The review adhered to the Preferred Reporting Items for Systematic Reviews and Meta-Analyses (PRISMA) standards. Given that this was a systematic review and the level of heterogeneity among the included studies was within an acceptable range, a meta-analysis was appropriately and rigorously conducted.

The eligibility criteria for this systematic review included women of reproductive and perimenopausal age group treated for AUB with either the drug ormeloxifene, a SERM, or norethisterone, a synthetic progesterone. The outcome measures were improvement in hemoglobin, endometrial thickness, and menstrual blood loss using the PBAC score. This review included a randomized controlled trial and a prospective comparative study where two groups were randomly assigned to receive either ormeloxifene or norethisterone. Only studies published in English were considered.

For the exclusion criteria, studies that investigated AUB due to structural causes were excluded, as were those addressing non-structural causes other than ovulatory dysfunction (AUB-O). Additionally, case series, studies with incomplete or insufficient data, and animal studies were not considered for inclusion.

Search Strategy

A comprehensive and systematic literature search was conducted from March 10, 2025, for three months using several databases. Using Medical Subject Headings (MeSH) terms, pearl growing, Boolean operators, and title search as search strategies for the PubMed database; citation chasing, author search, and title search for Google Scholar; and search strategies such as pearl growing, basic search with keywords, advanced search with Boolean operators, and field tags for the Web of Science database, as well as MeSH terms, Cochrane Search Manager, and search filters for Cochrane Library databases were used for literature search related to AUB. Keywords used were “ormeloxifene”, “norethisterone”, and “AUB-O”. Furthermore, a manual search of the reference list was conducted from the designated area of research, and appropriate publications were incorporated into the research analysis and literature review. Randomized controlled trials and prospective studies evaluated ormeloxifene and norethisterone, with outcomes such as improvement in hemoglobin, endometrial thickness, and PBAC score.

Study Selection

The studies were selected by entering the search results into Rayyan (Rayyan Systems Inc., Cambridge, MA), an online platform for systematic reviews. Two authors (S.D.L., H.V.) did the literature search and selected the potential studies by screening the titles, abstracts, and keywords of all studies. Two authors independently reviewed abstracts and complete studies to substantiate that the publications fulfilled the inclusion and exclusion criteria. Any conflicts or disagreements that developed throughout the selection process were resolved by consensus or adjudication by the third author (R.M.).

Data Extraction

The authors of this study independently collected important study characteristics for the review. A predetermined standardized checklist was used to extract data, which included the first author’s last name, publication year, total sample size, gender, study design, participant age, and type of intervention (ormeloxifene vs. norethisterone in AUB-O), as well as primary and secondary outcomes analyzed in the study (Table 1).

**Table 1:** Characteristics of study population ITT, intention-to-treat analysis; NES, norethisterone; PP, per-protocol analysis; RCT, randomized controlled trial

First author	Year of publishing	Study setting	Study design	Blinding	Study period	Age in years	Intervention group	Type of comparator	Type of analysis
Fatima et al. [9]	2024	Hospital	Prospective comparative study	Not reported	12 months	30 to 50	Ormeloxifene	NES	PP
Vardaini et al. [14]	2020	Hospital	Prospective comparative study	Not reported	Three months	25 to 45	Ormeloxifene	NES	ITT
Karmakar et al. [10]	2016	Hospital	RCT	Not reported	24 months	30 to 50	Ormeloxifene	NES	ITT
Srinivasan et al. [6]	2022	Hospital	RCT	Not reported	12 months	19 to 45	Ormeloxifene	NES	ITT
Meena et al. [1]	2022	Hospital	Prospective comparative study	Not reported	24 months	35 to 52	Ormeloxifene	NES	ITT

The obtained data were entered into Microsoft Excel (Microsoft Corporation, Redmond, WA) and verified again to remove any possible errors by the second and third authors. The obtained data were imported into the software RevMan 5.4 (The Cochrane Collaboration, Oxford, UK) by the first author (S.P.S.).

Quality Assessment

The selected publication’s risk of bias was assessed using the method of the revised Cochrane Risk of Bias for randomized and prospective studies. Thus, the quality of studies was monitored, and the studies were categorized as “low risk,” “some concerns,” and “high risk” of bias.

Statistical Analysis

RevMan 5.4 was used to do this meta-analysis. Notable variations seen across the studies were patients’ ages, duration of study, and follow-up. In methodology, the main difference noted was study design, which resulted in employing a logistic-normal random-effects model. Heterogeneity was assessed using I² statistics, and heterogeneity across the studies with I² > 50% or a p-value < 0.05 was considered clinically significant. Because of the small number of studies, a funnel plot or Egger’s test could not be used.

Results

Characteristics of the Study Population and Study Selection

**Figure 1:**
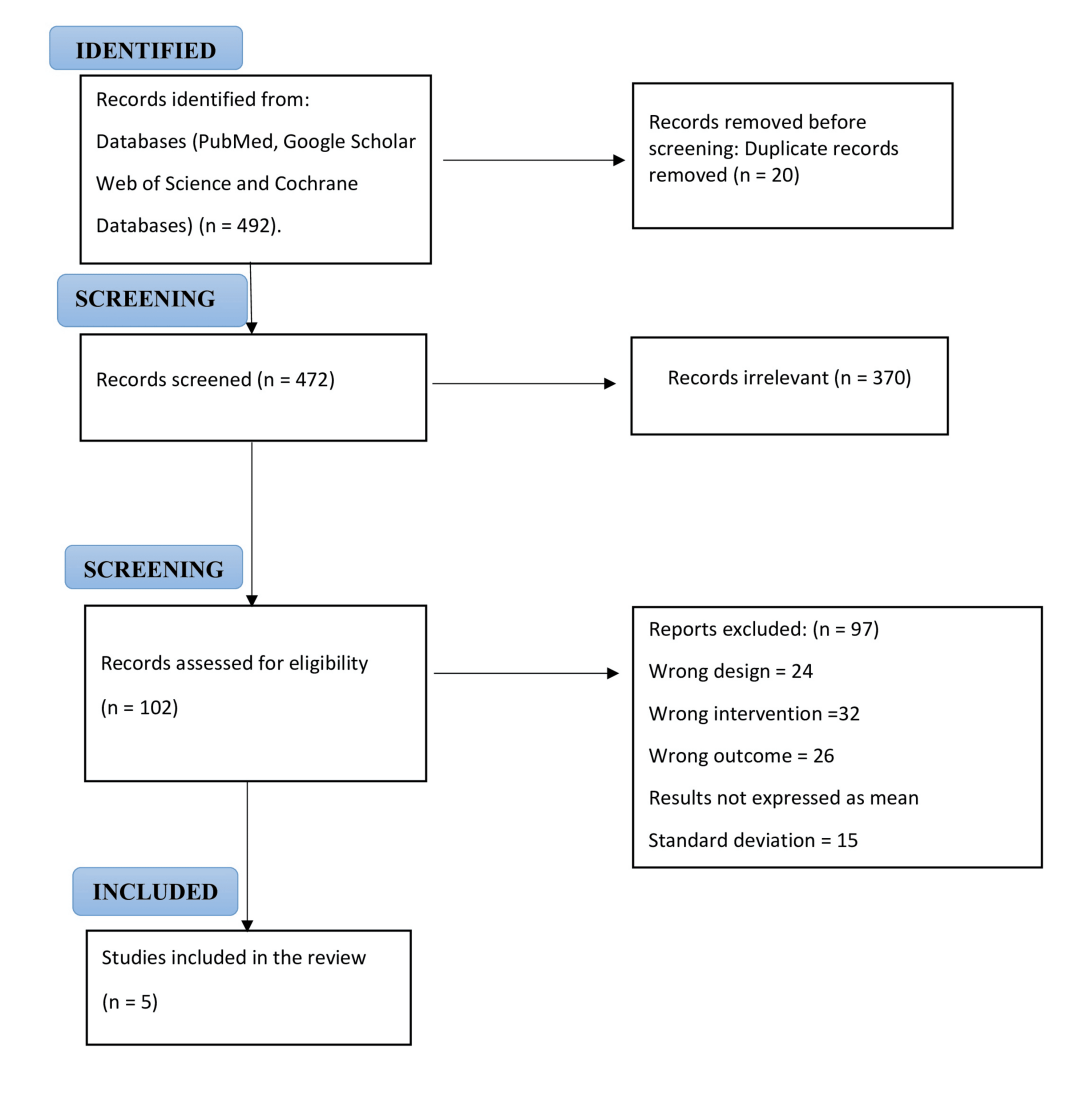
Preferred Reporting Items for Systematic Reviews and Meta-Analyses (PRISMA) flow diagram of study selection process

A total of 492 studies were initially retrieved following the removal of duplicates. On screening, 390 studies were deemed irrelevant to our review. The remaining 102 were assessed for eligibility. Of those, five studies met the inclusion criteria and were included for qualitative and quantitative analysis. Figure 1 illustrates the PRISMA flowchart for the study selection.

Methodological Quality of Included Studies

**Figure 2:**
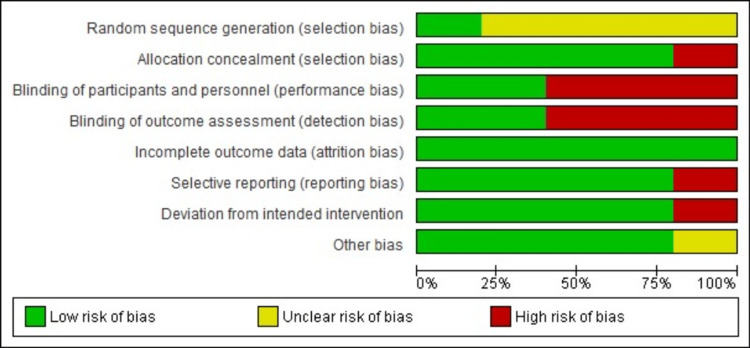
Risk of bias assessment graph

**Figure 3:**
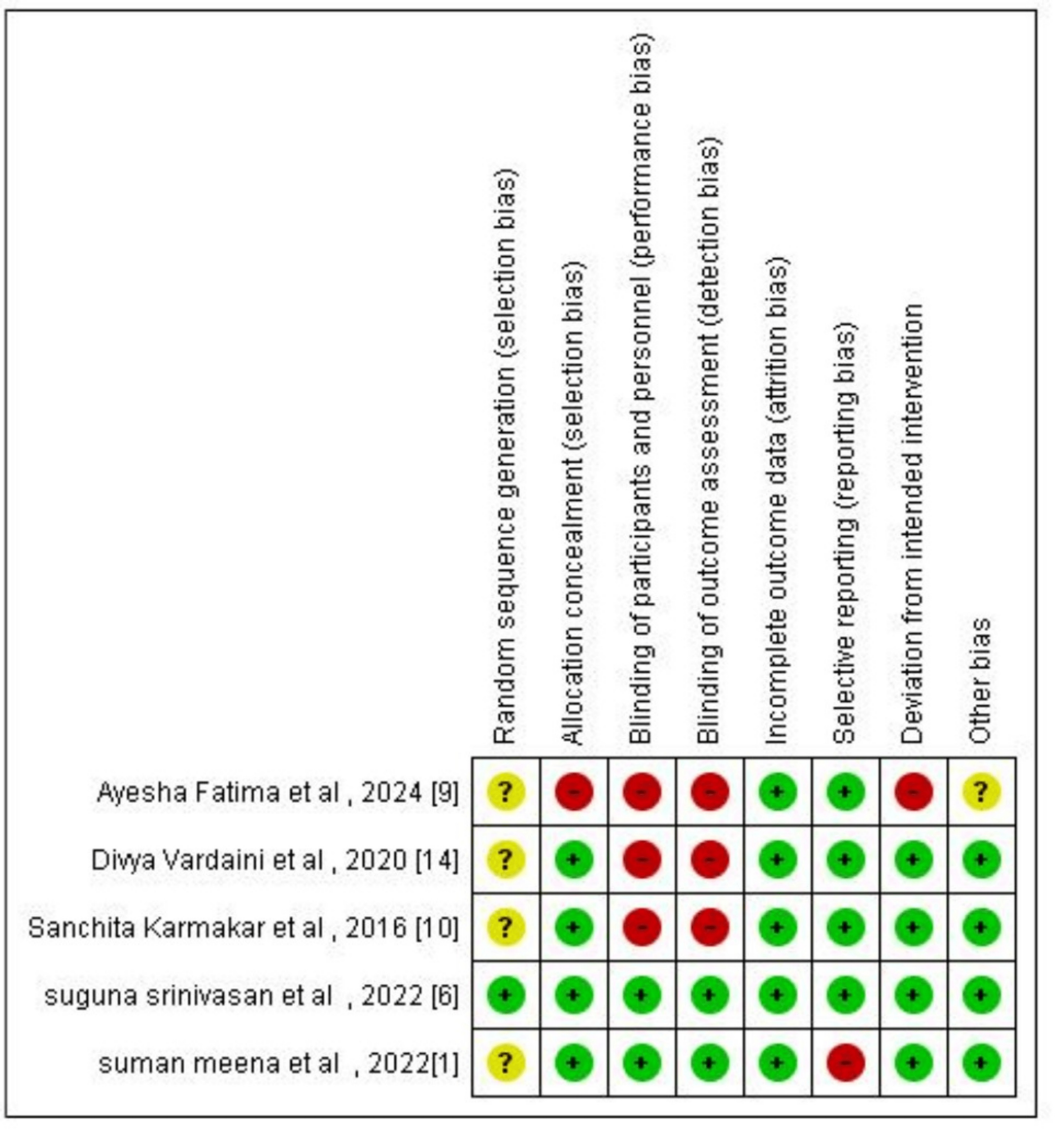
Risk of bias summary graph

Risk of bias assessment was performed using the RoB 2 tool (Figures 2-3) [15]. Allocation concealment was not done in one study; three out of five studies did not mention blinding, and deviation from the intended intervention was noted in one study. These articles were published in a hospital setting between 2016 and 2024. The findings analyzed were noted in appropriate sequence, starting with the research method of meta-analysis comparing ormeloxifene vs. norethisterone in AUB-O. The final review comprised two RCTs and three observational prospective studies with the drug norethisterone as the control.

**Figure 4:**
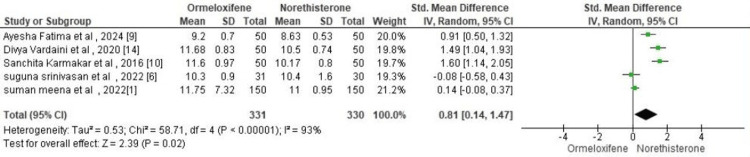
Forest plot comparing hemoglobin level in both groups

Our meta-analysis demonstrated that norethisterone is more effective than ormeloxifene in improving hemoglobin levels in patients with AUB-O. A total of five studies comprising 661 patients were included, with 331 patients in the ormeloxifene group and 330 in the norethisterone group. The pooled analysis showed a significant benefit in favor of norethisterone with a standardized mean difference (SMD) of 0.81 (95% CI: 0.14, 1.47); p = 0.02, indicating improved hematological outcomes. However, there was substantial heterogeneity among the included studies (I² = 93%, p < 0.00001). The observed heterogeneity could be attributed to differences in study design, sample size, and duration of treatment across trials. To explore this further, sensitivity analysis was performed, which showed that no individual study had a disproportionate influence on the overall effect size. Though the forest plot shows results favoring norethisterone, the SMD (green square) in the forest plot is almost or near zero, with the combined weight pulling towards norethisterone. Thus, although norethisterone appears superior, it does not differ to a great extent in efficacy compared with ormeloxifene. This might be due to the fact that, although ormeloxifene reduces bleeding to a great extent, it produces clumpy clots in a few patients, which might be the reason for the forest plot favoring norethisterone. This can be overcome by adding antifibrinolytics with ormeloxifene, which might reduce the occurrence of clots. Therefore, further studies are needed to evaluate the efficacy of ormeloxifene (Figure 4).

Statistician's Opinion 1

In the pooled analysis of five studies (n = 661), hemoglobin improvement was significantly greater in the norethisterone group compared with the ormeloxifene group. High heterogeneity (93%) likely reflects differences in study design, baseline Hb, and treatment duration. Sensitivity analysis showed no influential study, confirming the robustness of the effect.

**Figure 5:**
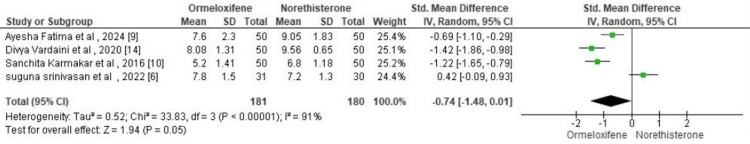
Forest plot comparing endometrial thickness in both groups

In Figure 5, four studies were compared involving 181 patients in the intervention group and 180 patients in the control group, which proved that ormeloxifene is better than norethisterone in reducing endometrial thickness (SMD = -0.74 with 95% CI (-1.48 to 0.01), p-value < 0.00001 with heterogeneity I² = 91%). This reduction in endometrial thickness is clinically significant, and it favors ormeloxifene over norethisterone. Sensitivity analysis did not show any uneven impact on the analysis and the result. Hence, heterogeneity in this comparison might be due to differences in duration of treatment and differences in sample size.

Statistician's Opinion 2

Four studies showed a significant reduction in endometrial thickness with ormeloxifene. High heterogeneity was expected due to variability in follow-up intervals and baseline measures. Direction of effect remained consistent.

**Figure 6:**
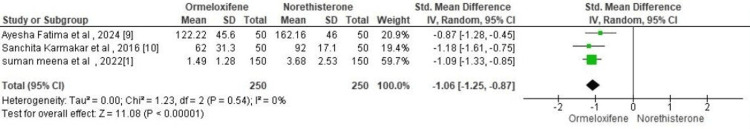
Forest plot depicting comparison of Pictorial Blood Assessment Chart (PBAC) score

Only three out of five studies compared PBAC scores, involving 250 patients in the intervention group and 250 patients in the control group (SMD = -1.06, 95% CI (-1.25, -0.87), with heterogeneity of I² = 0%; p-value (<0.00001), which is clinically significant). Thus, this analysis concludes that ormeloxifene is better than norethisterone in reducing blood loss, as demonstrated in Figure 6.

Statistician's Opinion 3

Three studies demonstrated a large and consistent reduction in PBAC scores favoring ormeloxifene. The absence of heterogeneity indicates highly uniform findings across studies.

Discussion 

AUB-O, being common in all stages of menstrual life, understanding the impact of AUB on quality of life can help healthcare providers develop an effective management plan that addresses both physical and emotional needs. The crucial part of managing AUB-O relies mostly on medical therapy. Although various medical methods are available, concerns remain regarding optimizing medical management, as AUB-O still has a profound impact on women’s health.

Ormeloxifene is the latest drug available for AUB. This SERM is a third-generation type that has affinity for ERs. It acts like estrogen in tissues including the vagina, bone, cardiovascular system, and central nervous system, with antiestrogenic effects on the uterus and breast [14]. This forms the basis for its role in the medical management of AUB-O. Also known as centchroman, it is nonhormonal and nonsteroidal and can be taken once or twice a week, which may improve patient compliance [16,17]. Norethisterone acts by suppressing endometrial development through an antiproliferative endometrial effect, thereby reducing menstrual abnormalities. Norethisterone is currently used as the first-line drug for HMB [16].

Several observational studies and randomized controlled trials [1,6,9,10,14] have compared ormeloxifene and norethisterone in AUB, demonstrating that ormeloxifene is more effective than norethisterone. Taking this into account, and with the aim of enhancing the provision of effective medical management, this systematic review and meta-analysis were conducted using five studies: two randomized controlled trials and three prospective comparative studies [1,6,9,10,14]. Our meta-analysis compared outcomes including hemoglobin, endometrial thickness, and PBAC score after treatment with ormeloxifene and norethisterone.

Analysis of this SRMA yielded results suggesting that ormeloxifene is better than norethisterone in reducing PBAC scores and endometrial thickness. Norethisterone is better than ormeloxifene in improving hemoglobin outcomes. As five studies were included for comparing hemoglobin and only three studies for comparing PBAC scores, patients in the additional two studies might have developed clots due to ormeloxifene, although this was not mentioned in the studies. This may explain the differences in outcomes for hemoglobin and PBAC scores. Various clinical trials have analyzed the effect of ormeloxifene in AUB-O. A study conducted by Chitrangada et al. [12], involving 100 patients, showed that ormeloxifene is better than norethisterone in treating AUB. A study by Chauhan et al. [18] demonstrated that ormeloxifene is better than norethisterone and OCPs in the management of AUB-O.

The study by GM et al. [19] concluded that ormeloxifene is better than norethisterone in improving hemoglobin. The study by HD et al. [20], involving 60 perimenopausal women, showed that ormeloxifene is better than norethisterone in reducing endometrial thickness. Both studies also demonstrated that PBAC scores were significantly reduced with ormeloxifene compared to norethisterone. A study by Jacob et al. [21] showed that ormeloxifene is better than norethisterone in reducing PBAC scores. Mainani et al. [22] demonstrated that ormeloxifene is superior to norethisterone in reducing PBAC, reducing endometrial thickness, and improving hemoglobin. Agarwal et al. [13] showed that parameters including hemoglobin, endometrial thickness, and PBAC scores improved more with ormeloxifene than with norethisterone. Almost all studies indicate that ormeloxifene is better than norethisterone in improving hemoglobin, reducing endometrial thickness, and reducing PBAC. Sulaiman et al. [23] demonstrated that ormeloxifene is more effective than conventional hormone therapies in reducing menstrual blood loss and endometrial thickness.

Strength of the Review

This is the first SRMA being conducted to analyze ormeloxifene vs. norethisterone in AUB-O. A thorough literature search using various databases has been conducted, which helps in synthesizing the results. Sensitivity and subgroup analyses were performed to reduce disproportionate results contributed by individual studies. By pooling data from various studies, this SRMA increases the statistical power to detect the effect of ormeloxifene in AUB-O.

Limitations or Drawbacks of the Review

Out of the 20 studies considered, only five were prioritized for meta-analysis, as the remaining studies did not report the standard deviations needed for statistical analysis. There is a lack of information regarding blinding in a few studies. The type of randomization was not mentioned in any of the studies, and the age groups included for analysis were not consistent across studies. Almost all studies originate from the same geographical region. There is a lack of studies from regions outside India, as ormeloxifene is a recently evolving drug for AUB, although it has been used in India as a birth control method since 1990 [24].

## Conclusions

The result of our systematic review and meta-analysis concluded that ormeloxifene is better than norethisterone in treating AUB-O. Ormeloxifene has the benefit of reducing endometrial thickness and blood loss compared to norethisterone, though hemoglobin improvement is greater with norethisterone. Also, considering the added benefits of ormeloxifene, including good patient compliance due to its long half-life and its nonhormonal, nonsteroidal effect, ormeloxifene proves to be better in the management of AUB-O.

On the other hand, there are a few reports by gynecologists that ormeloxifene produces clots resembling retained products of conception in some cases, though this does not affect most patients. Moreover, adverse effects were not analyzed or compared in these studies. More evidence-based observations or studies are required to evaluate its adverse effects, its impact on hemoglobin levels, and to help formulate further plans for promoting a healthier environment. Larger trials and international studies are also needed to improve the validation of observations in this SRMA and to apply the findings, which may bring a crucial change in the medical management of AUB-O.
